# Transcriptional regulation modulates nitrate uptake and utilization of *Pinus elliottii* under nitrogen stress

**DOI:** 10.3389/fpls.2026.1856478

**Published:** 2026-07-21

**Authors:** Shaoze Wu, Xianyin Ding, Qifu Luan, Xiahui Hua, Yadi Wu, Qinyun Huang

**Affiliations:** 1 Research Institute of Subtropical Forestry, Chinese Academy of Forestry, Zhejiang Key Laboratory of Forest genetics and breeding, Hangzhou, China; 2 College of Forestry and Grass/College of Soil and Water Conservation, Nanjing Forestry University, Nanjing, China

**Keywords:** nitrogen absorption, nitrogen stress, nitrogen utilization, *Pinus elliottii*, transcriptome

## Abstract

In natural and agricultural ecosystems, nitrogen deficiency is often a major limiting factor for plant growth, while research on the molecular genetic basis of nitrogen uptake and utilization in slash pine remains scarce. This study explored the regulatory mechanisms of pine needles under different conditions by comparing the differences in nitrogen response of needles from different slash pine families at different time points under varying nitrogen concentrations, aiming to better understand the expression profile of nitrogen uptake and utilization in slash pine needles. Three key results were obtained: (1) A total of 2353 differentially expressed genes were identified, mainly including transcription factors families, such as C3H, bHLH, MYB, and others. These genes have been reported to be associated with nitrogen uptake and utilization in plants. (2) The nitrogen uptake and utilization pathway contains 36 DEGs encoding 10 types of proteins. (3) Weighted Gene Co-expression Network Analysis showed a strong correlation between ground diameter of slash pine and module gene expression, and the hub gene *Pee07g026510* exhibited high connectivity with other nitrogen metabolism genes. This confirms that *Pee07g026510* can affect Diameter growth by regulating GAD expression—specifically, generating GABA to alleviate ammonium toxicity under high-nitrogen conditions and prioritizing the efficiency of the GS/GOGAT cycle under low-nitrogen conditions. This is consistent with the theory that nitrogen deficiency inhibits biomass accumulation, providing a fundamental reference for the management and genetic improvement of slash pine plantations.

## 1 Introduction

Nitrogen represents an essential macronutrient throughout the whole growth and developmental processes of plants. As a fundamental constituent of numerous biological macromolecules including proteins, nucleic acids, and chlorophyll, nitrogen participates extensively in diverse physiological activities, such as photosynthesis, biosynthesis, and metabolic regulation (O’Brien et al., 2016; Chen et al., 2021). In both natural and agricultural ecosystems, nitrogen deficiency frequently serves as a primary constraint restricting plant growth. However, excessive nitrogen fertilization leads to persistently low nitrogen use efficiency (NUE), typically ranging from 30% to 50% (Tilman et al., 2002), and also triggers serious environmental issues including soil acidification and water eutrophication. Therefore, exploring the molecular regulatory networks underlying plant nitrogen uptake and utilization, as well as breeding cultivars with improved nitrogen use efficiency, have become essential strategies for ecological conservation and sustainable industrial development (Zeigler and Mohanty, 2010).

As a nitrogen-containing pigment, chlorophyll synthesis directly depends on nitrate availability, which regulates its precursor production (e.g., glutamate) through nitrate reductase, nitrite reductase, glutamine synthetase, and glutamate synthase enzymes (Martin et al., 2006; Pinto et al., 2014). Nitrate deficiency impairs chlorophyll biosynthesis, disrupts chloroplast structure (thylakoid membranes), and accelerates degradation via pathways involving chlorophyllase and Mg-dechelatase, leading to decreased photosynthetic efficiency and plant growth (Tóth et al., 2002; Schelbert et al., 2009). Thus, monitoring chlorophyll levels provides key insights into nitrogen utilization efficiency, photosynthetic capacity, and physiological responses to nitrate treatments, enabling precise evaluation of treatment effects and optimizing management strategies for improved plant health and productivity.

To date, numerous studies have focused on the mechanisms underlying nitrogen uptake and nitrogen stress responses in various tree species. Under low-nitrogen and nitrogen-free conditions, the woody oil species Yellowhorn exhibits a large number of differentially expressed genes. Its adaptation to low-nitrogen stress mainly relies on the upregulation of pathways related to phenylpropane biosynthesis, flavonoid biosynthesis, and plant hormone signal transduction, among which the gibberellin, salicylic acid, and jasmonates signaling pathways serve key regulatory roles (Li et al., 2023). In *Eucalyptus grandis*, the NPF gene family participates in nitrogen uptake and allocation. Distinct nitrogen conditions significantly affect the expression of several EgNPF genes, which enhance tolerance to nitrogen stress by regulating nitrogen transport and redistribution (Li et al., 2024). Studies in poplar have demonstrated that nitrogen supply not only affects growth morphology and photosynthetic physiology, but also mediates drought acclimation and adaptation to nitrogen starvation or excess by modulating plant hormone signals, antioxidant systems, and carbon-nitrogen metabolic pathways, where global transcriptome reprogramming acts as the core molecular mechanism (Luo et al., 2015; Lu et al., 2019). These studies provide valuable references for revealing the molecular regulatory patterns of nitrogen responses in woody plants and further highlight the necessity of investigating nitrogen response mechanisms in *Pinus elliottii*.

*Pinus elliottii* Engelm var. elliottii (slash pine) is an important fast-growing afforestation species in southern China, valued for both timber and resin production, as well as a carbon sequestration species. It supports the development of multiple industries such as forest chemical engineering. However, nitrogen fertilizer loss is severe in pine forests in southern China, and nitrogen stress leads to poor growth of nearly 700000 hectares of slash pine, directly causing shortages of raw materials for related industries (Liu et al., 2020). Although genetic improvement of pine species has a history of nearly a century internationally, and China has carried out slash pine breeding for more than 40 years, existing studies mostly focus on the improvement of phenotypic traits such as growth and wood properties. Restricted by the large and conserved genome of slash pine and its long growth cycle, breeding efficiency remains low (Neale and Savolainen, 2004; Ding et al., 2022), while research on the molecular genetic basis of its nitrogen uptake and utilization is still insufficient.

Plant nitrogen uptake and utilization is a complex regulatory process. Existing studies have shown that plants absorb inorganic nitrogen through nitrate transporters (e.g., NRT1, NRT2 families) and ammonium transporters (AMT family), and complete nitrogen assimilation via key enzymes such as glutamine synthetase (GS) and glutamate synthase (GOGAT). This process is simultaneously regulated by multiple levels of factors including transcription factors and miRNAs (Ishiyama et al., 2004; Lothier et al., 2011; Krapp, 2015; Jung et al., 2023). Transcriptomics technology (RNA-seq), with its advantages of high efficiency and sensitivity, has played an important role in screening nitrogen-responsive genes in crops and some forest trees, successfully identifying structural genes and regulatory factors related to nitrogen metabolism (Wang et al., 2018; Zhang et al., 2023). However, in current studies on slash pine, there is no systematic analysis of dynamic temporal response differences among different families under varying nitrogen concentrations, and the key regulatory genes and molecular networks involved in nitrogen uptake and utilization remain unclear.

In view of this, this study used slash pine as the research material, focusing on the differences in nitrogen response of needles from different families at the seedling stage under different nitrogen concentration treatments. Combining time-series analysis and transcriptomics technology, it deeply explored the regulatory mechanisms of nitrogen uptake and utilization in slash pine and screened key regulatory genes. The objectives of this study were: (1) to determine the changes in transcriptional expression levels, nitrogen content, plant height, and ground diameter of slash pine needles under different nitrogen concentration treatments; (2) to analyze the expression of differential expressed genes (DEGs) in needles under different nitrogen stress conditions and screen DEGs related to nitrogen uptake and utilization; (3) to screen hub genes associated with various components using weighted correlation network analysis (WGCNA) and analyze the relationship between phenotypes (e.g., plant height, ground diameter) and gene expression.

## 2 Materials and methods

### 2.1 Plant materials and experimental treatment

To determine the optimal nitrogen application concentration for slash pine, prior studies provide valuable reference. Dewald et al. tested nitrogen concentrations ranging from 1 to 21 mmol·L^-1^ in slash pine and identified 13 mmol·L^-1^ as the optimal level for growth promotion (Dewald et al., 1992). In contrast, Lin et al. treated Cunninghamia lanceolata with sodium nitrate at concentrations of 2, 5, 10, and 15 mmol·L^-1^, observing an initial increase in root growth rate followed by a decline (Lin et al., 2024). This non-linear response indicates that the optimal concentration identified by Dewald (13 mmol·L^-1^) aligns with the transition point where root growth begins to plateau in Lin’s experimental range. Similarly, Zhu et al. applied nitrogen at 0, 5, 10, and 15 mg per plant to Larix olgensis, and the growth rates of stems and roots also exhibited a trend of increasing first and then decreasing (Zhu et al., 2013). Furthermore, Zhu et al. reported that stem biomass accumulation was relatively slow during 2–6 weeks of nitrogen treatment. Based on the above-mentioned nitrogen application regimes in conifers and *Pinus elliottii*, combined with our preliminary experiments, calcium nitrate was used as the nitrogen source in this study. Three treatments were established, the concentration of calcium nitrate in the three groups is: a high-nitrogen group (30 mmol·L^-1^), a low-nitrogen group (10 mmol·L^-1^), and a no-nitrogen control (0 mmol·L^-1^). Designating the group without nitrogen application as the control group serves to exclude the influence of other factors besides nitrate on the experimental outcomes. The test materials were composed of seedlings from 30 half sib families of slash pine (Diao et al., 2024).

In March 2024, 6-month-old light substrate non-woven container seedlings were transplanted into pots (top diameter: 23.0 cm, bottom diameter: 21.5 cm, height: 21.0 cm) filled with 5 kg of red soil. The total nitrogen and total phosphorus contents of the red soil were 54 mmol·L^-1^ and 3 mmol·L^-1^, respectively. For each family, 9 seedlings of uniform height and ground diameter were selected for transplantation and arranged in a row. Every 3 seedlings were assigned to one treatment, corresponding to the three nitrogen concentration settings. In total, 270 seedlings were used across the 30 families. After transplantation, the seedlings were acclimated for 2 months under natural temperature and light conditions in the nursery. For the nitrogen treatment, Hoagland’s nitrogen-free nutrient solution was used as the stock solution. Calcium nitrate was added to the stock solution to achieve the target concentrations for the high-nitrogen and low-nitrogen groups, while no calcium nitrate was added to the control group. The prepared solution was poured into the pots of the corresponding treatment groups until the solution drained out from the bottom of the pots. This experiment was conducted in the nursery of Changle Forest Farm, Yuhang District, Hangzhou City, Zhejiang Province, China (119°50’E, 30°20’N).

The seed source of slash pine seedlings originated from the clonal seed orchard of Changle Forest Farm (a state-owned forest farm) in Hangzhou, Zhejiang Province. This seed orchard was established in 2011 and consists of 49 elite clones, which were selected by experienced breeders from tens of thousands of slash pine commercial forests in the region based on their superior growth and resin production traits. These elite trees have been genotyped using SNP-array technology, providing queryable genotype information (Diao et al., 2024). In 2023, seeds from 30 open-pollinated families were randomly collected from the seed orchard according to clone, and seedlings were cultivated. The experiment was initiated in spring 2024. The nitrogen treatment was initiated on April 21, 2025, with needle samples collected from the same plants at two time points: the first sampling occurred on April 24, 2025 (3 days after treatment initiation), yielding 269 samples, and the second sampling took place on May 12, 2025 (21 days after treatment initiation) (Zhang et al., 2014; Mozo et al., 2025), yielding 270 samples, resulting in a total of 539 samples, with the missing sample attributed to the low-nitrogen group of family 26 during the 3 days collection. In total, 539 samples were collected. All collected samples were stored at -80°C for subsequent experimental analysis.

### 2.2 Statistical analysis of phenotypic data

Analysis of variance was performed using the mixed linear model implemented in ASReml software to evaluate differences and descriptive statistics of growth traits in *Pinus elliottii* at the treatment and family levels. The statistical model was defined as follows:


$$\text{Y}={\text{X}}_{1}\text{b}+{\text{X}}_{2}\text{f}+{\text{X}}_{3}\text{d}+\text{e}$$

where Y is the vector of phenotypic observations; X_1_, X_2_, and X_3_ represent the incidence matrices for treatment, family, and sampling time, respectively; b, f, d, and e represent the vectors of fixed treatment effects, family effects, time effects, and random residual errors, respectively.

### 2.3 RNA extraction, sequencing and analysis

In this study, total RNA was extracted from pine needle samples using the RNAprep Pure Plant Plus Kit (Cat. No. DP441, Tiangen Biotech, Beijing, China). RNA purity and integrity were examined using a NanoDrop2000 spectrophotometer (Thermo Fisher Scientific, USA) to ensure qualification for downstream experiments. PolyA-tailed mRNA was enriched using oligo-dT magnetic beads, followed by library construction and RNA-seq analysis. Libraries with valid concentrations ≥ 2 nM were sequenced on an Illumina NovaSeq 6000 platform at Jizhigene Technology Co., Ltd. (Tianjin, China) using paired-end sequencing. Raw sequencing data contained adapter sequences and low-quality reads. Raw data were initially processed in FASTQ format, and TrimGalore (v0.4.5) was used to remove adapter sequences, reads containing ambiguous ‘N’ bases, and low-quality reads, yielding clean reads for further analysis. Q20 and Q30 values were calculated to evaluate data quality. High-quality clean data were used for downstream analyses. Raw sequencing data generated in this study were deposited in the China National Center for Bioinformation (CNCB) under the accession number PRJCA052915 (https://www.cncb.ac.cn/). Clean reads were mapped to the *Pinus elliottii* reference genome downloaded from the TreeGenes database (https://treegenesdb.org/jbrowse?page=1) using HISAT2 (v2.0.5). Gene expression levels were calculated as fragments per kilobase of transcript per million mapped reads (FPKM) (Khan et al., 2026; Xu et al., 2026) using FeatureCounts (v1.5.0p3). Differentially expressed genes (DEGs) between the control and treatment groups were identified using the DESeq2 package (v1.20.0) in R(q<0.05 and |log2(Fold Change, FC)|> 1) (Wang et al., 2010).

### 2.4 Functional annotation and enrichment analysis

Protein sequences of DEGs were uploaded to the EggNOG v5.0 database (http://eggnog5.embl.de/#/app/home) for sequence alignment and functional annotation. The clusterProfiler (Yu et al., 2012) package in R (v4.5.1) was used to integrate and visualize Gene Ontology (GO) enrichment and pathway enrichment analyses at each stage. Expression levels of DEGs involved in nitrogen uptake and utilization were normalized by log_2_(FPKM + 1) and presented as heatmaps.

### 2.5 Phenotypic determination

**Figure 1:**
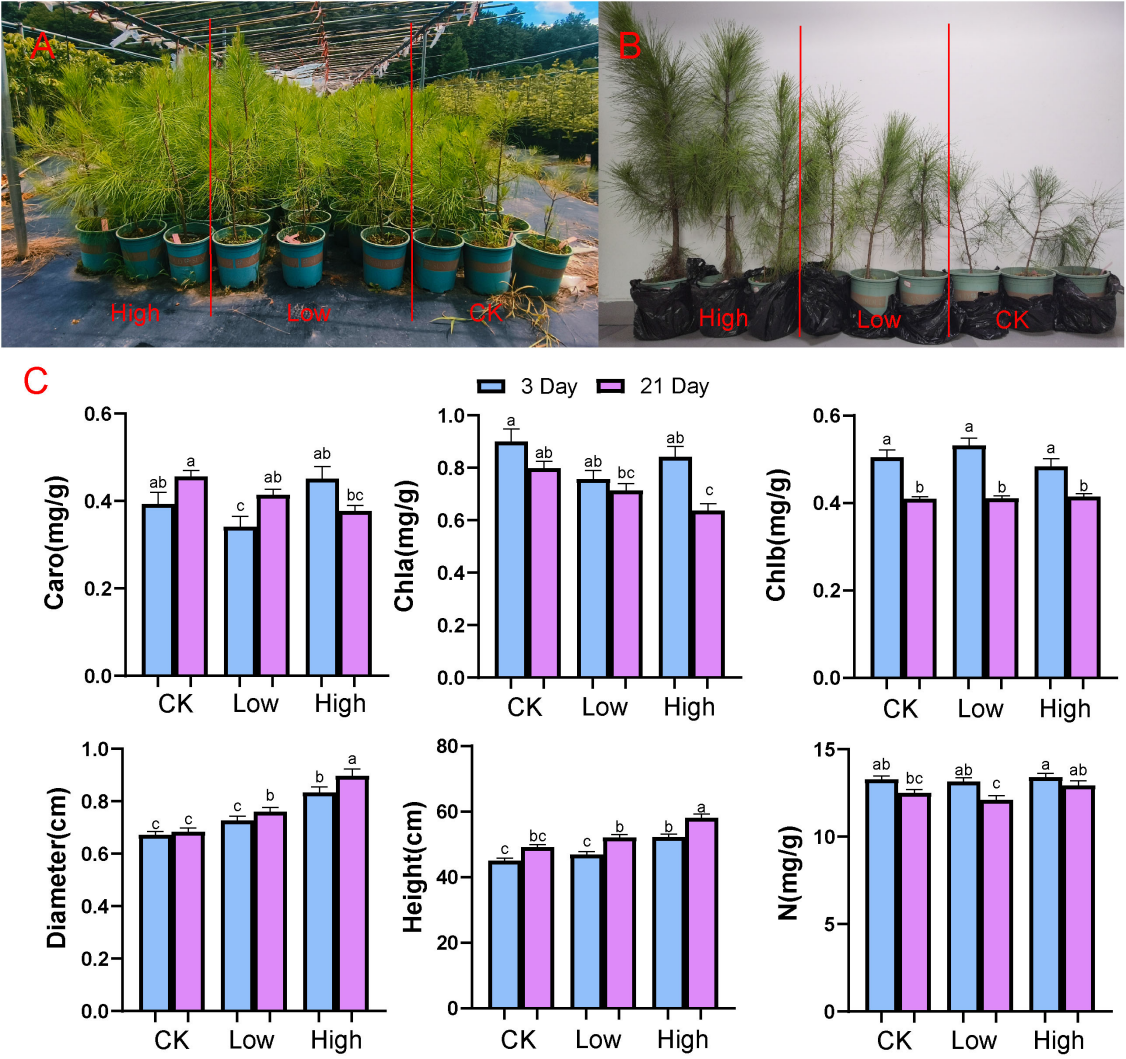
Growth status of slash pine seedlings and determined phenotypic analysis. **(A)** Growth status of all potted slash pine seedlings in the experiment, arranged from left to right as the high-nitrogen, low-nitrogen, and control groups. **(B)** Nine seedlings selected from the three groups (arranged from left to right as high-nitrogen, low-nitrogen, and control groups) for the determination of malondialdehyde, proline, and reactive oxygen species. **(C)**Blue bars represent phenotypic values sampled on day 3, and red bars represent those sampled on day 21. From left to right are the control group, high-nitrogen group, and low-nitrogen group. Different lowercase letters in the column **(A–C)** signify significant differences between two varieties under various treatments for the same index (*p* < 0.05).

Nitrogen content determination: The nitrogen content of the samples was measured using a continuous flow injection analyzer (Model: YLSZJ-SB-294).Seedling height and ground diameter determination: The ground diameter and height of each seedling were measured with a diameter tape during the two sampling campaigns. Chlorophyll content determination: The chlorophyll content in the needles of the 539 samples was determined using the Arnon method (Arnon, 1949).The growth performance of slash pine (*Pinus elliottii*) is shown in **Figure 1A**. Three seedlings were selected from each of the high-nitrogen, low-nitrogen, and control groups, as illustrated in **Figure 1B**. The contents of malondialdehyde (MDA), proline (Pro), and reactive oxygen species (ROS) in the roots, stems, and leaves of each seedling were determined. The contents of proline, malondialdehyde, and reactive oxygen species were measured using commercial assay kits manufactured by Suzhou Grace Biotechnology Co., Ltd. The corresponding catalog numbers of the kits were G0111W48 (Pro), G0109W48 (MDA), and GY0163W48 (ROS), respectively.

### 2.6 Weighted gene co-expression network analysis

Weighted gene co-expression network analysis has been widely used in bioinformatics studies to identify gene modules and hub genes (Smita et al., 2013). Based on genome-wide gene expression profiles of 539 *Pinus elliottii* tissue samples, a weighted gene co-expression network was constructed using the WGCNA package in R (Langfelder and Horvath, 2008). Transcriptome data were normalized, and FPKM values of 7000 genes from the 539 samples were retained for network construction. Genes with FPKM > 1 were screened from the differentially expressed genes identified across the 539 samples. Pearson correlation analysis was then performed between these screened genes and needle nitrogen content, and the top 10,000 genes with the highest absolute correlation coefficients were selected. These selected genes were further subjected to Pearson correlation analysis with ground diameter, and the top 7,000 genes ranked by absolute correlation coefficient were chosen for WGCNA analysis. A soft threshold power of 7 was selected to generate co-expression modules. Module–trait relationships were analyzed to identify modules highly correlated with phenotypic traits. Key modules of interest were determined, and hub genes related to nitrogen uptake and utilization were screened according to the criteria of module membership > 0.8 and gene significance > 0.2 (Zhu et al., 2021; Zhang et al., 2025). Hub genes were extracted from the target modules, and the eigengene network was visualized using Cytoscape (v3.9.2) (Shannon et al., 2003).

### 2.7 Real-time quantitative PCR

To verify the transcriptome results, 10 DEGs involved in nitrogen uptake and utilization were selected for expression validation. Nine samples with the largest ground diameter and nine samples with the smallest ground diameter were used for RT-qPCR analysis. Specific primers were designed using SPDE 2.0 (Xu et al., 2020). First-strand cDNA was synthesized from total RNA using a reverse transcription kit (Takara, 036 A). The diluted cDNA product was used as the template for RT-qPCR amplification. Relative gene expression levels were calculated using the 2^-^ΔΔCT method (Rieu and Powers, 2009). The UBI gene was used as the internal reference gene throughout the experiments (De Lima et al., 2016).

## 3 Results

### 3.1 Phenotype and correlation analysis

Measurements of needle nitrogen (N) content, chlorophyll a (Chla), chlorophyll b (Chlb), carotenoid (Caro) content, plant height (Height), and ground diameter (Diameter) in slash pine seedlings are shown in **Figure 1C**. Carotenoid content at day 3 followed: high nitrogen group > control group > low nitrogen group, while at day 21, the order was control group > low nitrogen group > high nitrogen group. Chlorophyll a content at day 3 was: control group > high nitrogen group > low nitrogen group, and at day 21: control group > low nitrogen group > high nitrogen group, with overall higher levels at day 3 than day 21. Plant height and ground diameter showed consistent trends at both day 3 and day 21: high nitrogen group > low nitrogen group > control group, with values at day 21 generally higher than at day 3. Nitrogen content was higher at day 3 than day 21. At day 3, differences among treatments were minimal, whereas at day 21, the control and high nitrogen groups showed similar levels, both higher than the low nitrogen group.

Phenotypic traits differed significantly among families, treatments, and sampling times. The results of ANOVA are presented in **Table 1**. The effects of family and treatment were highly significant for all phenotypic traits except chlorophyll b. Sampling time also exerted highly significant effects on all traits except carotenoids.

**Table 1:** Analysis of variance results of phenotype.

Character	Impact factors		
	Family	Treatment	Time
Diameter (mm)	2.8817***	59.8281***	6.5859*
Height (cm)	3.5048***	49.8274***	56.2824***
N (g/kg)	1.5825*	3.0307*	17.4795***
Chla (mg/g)	4.2261***	8.4349***	20.7166***
Chlb (mg/g)	3.6009***	1.7034	97.0798***
Caro (mg/g)	4.4994***	3.2267*	1.711

Phenotypes with significant levels of *p* < 0.05 were marked with “*”, those with significant levels of *p* < 0.01 were marked with “**”, and those with significant levels of *p* < 0.001 were marked with “***”.

**Figure 2:**
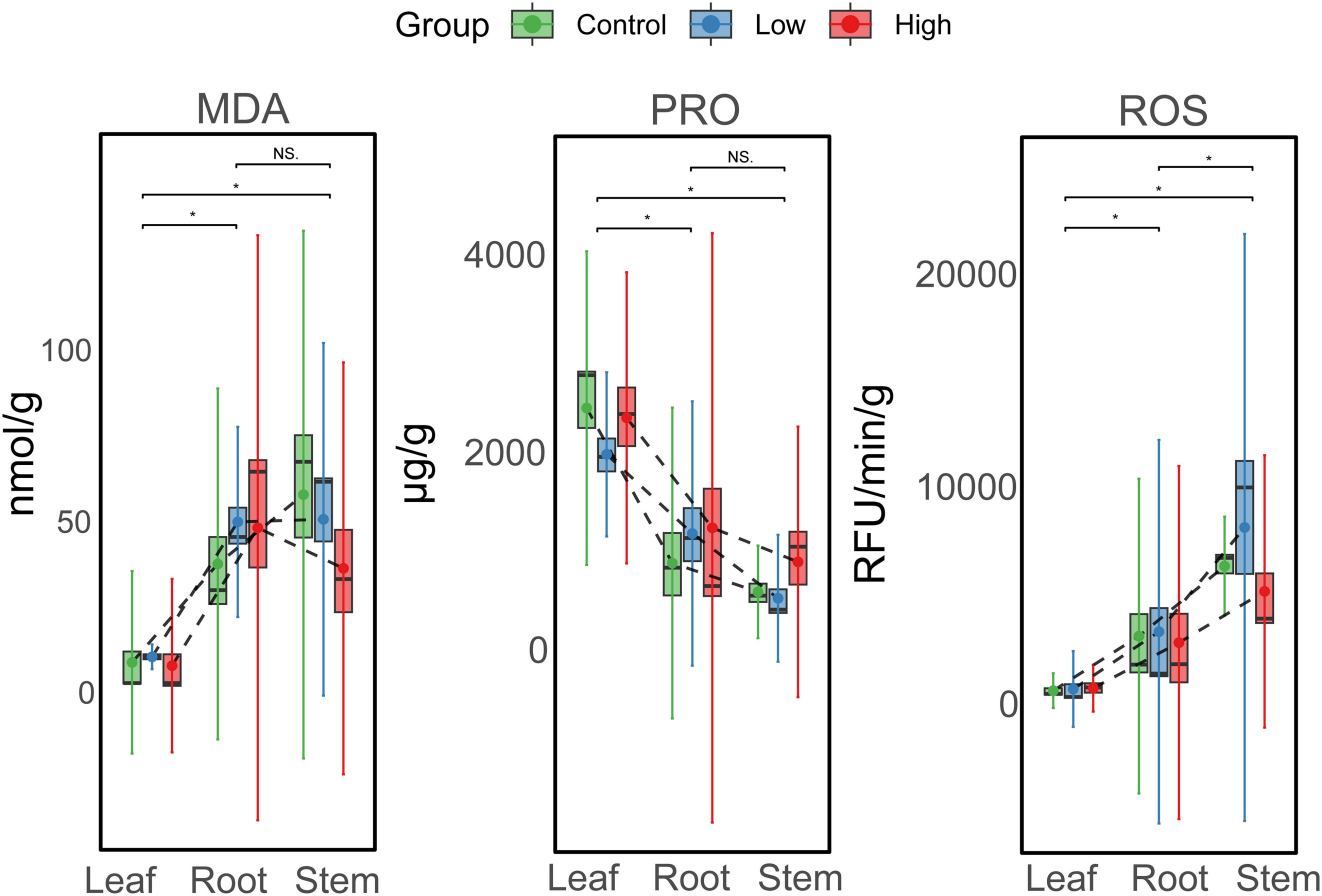
Analysis of stress indicators. Green represents the control group, red represents the high-nitrogen group, and blue represents the low-nitrogen group.”*” indicates a significance level of *p* < 0.05, “NS” indicates a significance level of *p* > 0.05.

To demonstrate the stress experienced by slash pine under low-nitrogen conditions using physiological indicators. The determination results of three stress indicators in the roots, stems, and leaves of *Pinus elliottii* seedlings are shown in **Figure 2**. The reactive oxygen species (ROS) content was lowest in all organs (roots, stems, and leaves) under the high-nitrogen treatment, indicating that both the low-nitrogen and control groups indeed experienced stress conditions. Proline content was elevated in roots and stems, which may be attributed to the higher nitrogen availability promoting active amino acid synthesis, thereby increasing proline accumulation in these tissues. Malondialdehyde (MDA) content was lowest in leaves and stems under the high-nitrogen treatment, which is consistent with expectations. However, MDA content in roots was higher in the high-nitrogen group compared to the other two groups, possibly due to accelerated root growth and subsequent senescence, leading to greater MDA accumulation.

### 3.2 Transcriptome sequencing data

In total, 539 sequencing libraries were sequenced on the Illumina NovaSeq platform, generating 3845.64 Gb of clean data. The average Phred Quality Score (Q30) value across all libraries was 97.25%, indicating high-quality data suitable for further analysis. Approximately 93.16% of the clean reads were successfully mapped to the reference genome, meeting the requirements for bioinformatic analysis.

### 3.3 Screening of DEGs under different nitrogen application conditions

**Figure 3:**
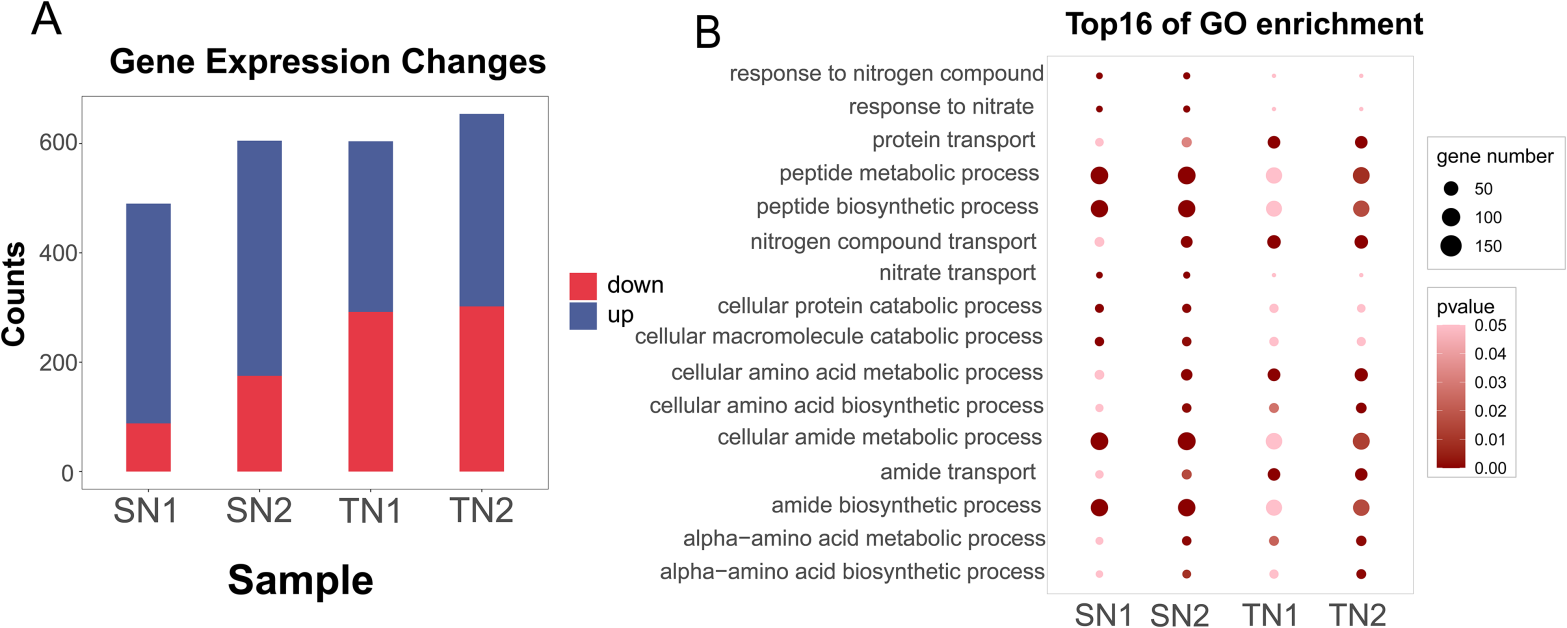
The differential expression patterns and top 16 significantly enriched GO terms of DEGs under different nitrogen concentrations and time points. **(A)** Comparison of paired DEGs in needles between each treatment group and its corresponding control group at the same sampling time point. **(B)** Comparison of GO terms enriched by DEGs under different nitrogen levels and treatment durations. Only the top 16 enriched GO terms (*p* < 0.05) are displayed. Circle size represents the number of DEGs, and color reflects the *p*-value: darker red indicates a smaller p-value and greater enrichment significance. SN1, SN2, TN1, and TN2 represent low nitrogen for 3 d, high nitrogen for 3 d, low nitrogen for 21 d, and high nitrogen for 21 d, respectively.

A comparative analysis of needles from treatment and control groups under varying nitrogen concentrations identified a total of 2353 differentially expressed genes (DEGs) (**Figure 3A**) (**Supplementary Data Sheet 1**). The number of DEGs was significantly higher at 21 days than at 3 days across all groups. Furthermore, the high-nitrogen group exhibited a significantly greater number of DEGs compared to the low-nitrogen group. At the 21-day time point, the number of down-regulated DEGs was greater, while the number of up-regulated DEGs was smaller, relative to the 3-day time point.

All DEGs were functionally annotated using the Gene Ontology (GO) database. GO enrichment analysis revealed significant enrichment in biological processes including response to nitrate and its transport, cellular amide biosynthesis, transport and metabolism, response to and movement of nitrogen-containing compounds, cellular amino acid biosynthetic and metabolic processes, and alpha-amino acid biosynthetic metabolism, among others (**Figure 3B**). Among the DEGs enriched in biosynthetic pathways, the majority were associated with C3H, bHLH, MYB-type transcription factors, nitrate and ammonium transporters, amidotransferases, as well as amino acid-related dehydrogenases, lyases, and synthases. This study focuses specifically on the DEGs involved in the nitrate absorption and utilization pathway.

### 3.4 Regulation of DEGs in the nitrogen uptake and utilization pathway

**Figure 4:**
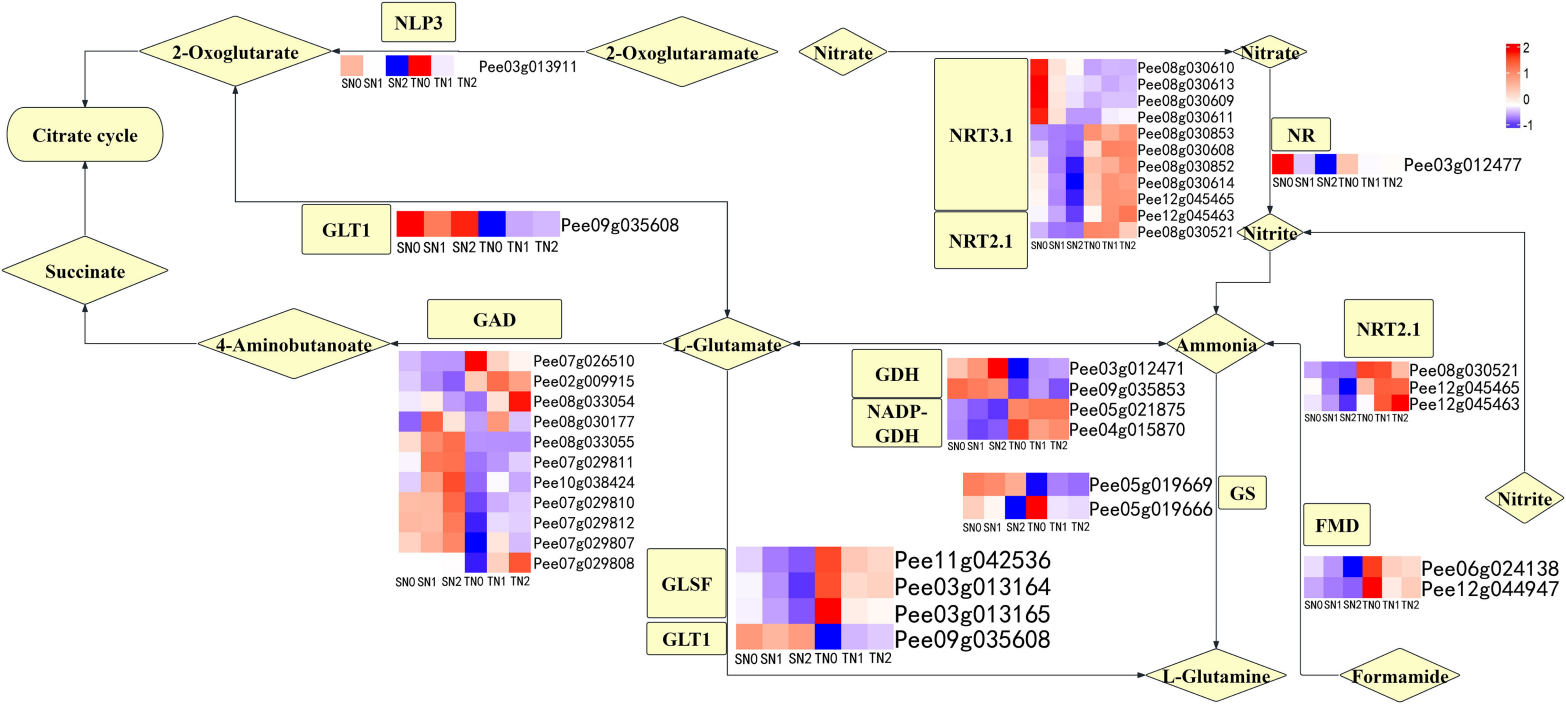
Heatmap showing the expression patterns of 36 DEGs involved in nitrate utilization and synthesis pathways. Rectangles represent key enzyme-encoding genes in these pathways, and diamonds represent substrates or reactants. Gene expression levels were normalized as log_2_(FPKM + 1) based on RNA-seq data. The color scale from blue to red indicates the expression level: darker red represents higher expression.

To elucidate the molecular regulation of nitrate utilization in slash pine under different nitrogen regimes, we further analyzed the role of DEGs in the nitrate uptake and utilization pathway. Within the core processes of nitrate utilization, a total of 36 DEGs were identified. These primarily included genes encoding three key protein types: high-affinity nitrate transporters (NRTs), nitrate reductase (NR), ferredoxin-dependent glutamate synthase (GLSF), glutamate synthase (GLT), glutamate dehydrogenase (GDH),NADP-specific glutamate dehydrogenase (NADP-GDH), Glutamine synthetase (GS), glutamate decarboxylase (GAD), Omega-amidase (NLP3) and formamidase (FMD). These results indicate that soil nitrogen concentration regulates the absorption and utilization of nitrate in slash pine at the molecular level. The expression patterns of these 36 DEGs, transformed using log2(FPKM + 1), are presented in **Figure 4**. The results demonstrate distinct expression profiles between treatment and control groups under different nitrogen concentrations and at different time points.

Within the nitrate assimilation pathway, among the 36 genes screened, several genes associated with nitrate uptake and reduction showed significant variations under different nitrogen treatments and time points. The uptake of extracellular nitrite is regulated by nitrate transporter 2.1 (NRT2.1). After 3 days of treatment, NRT2.1 gene expression was progressively suppressed with increasing nitrogen supply. By day 21, the expression of genes *Pee12g045465* and *Pee12g045463* increased with higher nitrogen levels, whereas gene *Pee08g030521* exhibited a decreasing trend. Nitrate uptake-related genes are also involved in extracellular nitrate absorption. The genes associated with NRT2.1 (*Pee12g045465, Pee12g045463* and *Pee08g030521*) and nitrate transporter 3.1 (NRT3.1) (*Pee08g030852, Pee08g030614, Pee08g030853* and *Pee08g030608*) displayed consistent expression patterns across nitrogen treatments and time points, suggesting potential coordinated action in nitrate transport, which aligns with previous studies. Other NRT3.1 genes (*Pee08g030610, Pee08g030613, Pee08g030609* and *Pee08g030611*) showed significantly higher expression in the control group at day 3 compared to other treatments and time points, indicating their important role in nitrate uptake under low nitrogen stress. The nitrate reductase gene (*Pee03g012477*) showed significant differences among treatments as early as 3 days after nitrogen application, with these differences largely diminishing by day 21, demonstrating its rapid responsiveness to nitrogen concentration changes. Its expression decreased with increasing nitrogen supply, further supporting its functional importance under low nitrogen conditions. Another nitrogen-responsive step in the nitrate assimilation pathway is the conversion of formamide to ammonia catalyzed by formamidase. The expression levels of formamide enzyme genes (*Pee06g024138* and *Pee12g044947*) were significantly lower at 3 days of treatment than at 21 days, and decreased with increasing nitrogen concentration at all time points.

In the glutamate metabolic pathway, glutamine synthetase gene (*Pee05g019669*) was highly expressed at day 3 but decreased by day 21, with a significant reduction in the control group at day 21. In contrast, another glutamine synthetase gene (*Pee05g019666*) showed significantly decreased expression under high nitrogen stress at day 3, while its expression increased significantly in the control group by day 21. Glutamate dehydrogenase genes (*Pee03g012471* and *Pee09g035853*) were up-regulated at day 3 and down-regulated at day 21. Conversely, NADP-glutamate dehydrogenase genes (*Pee05g021875* and *Pee04g015870*) were down-regulated at day 3 and up-regulated at day 21. Ferredoxin-dependent glutamate synthase genes (*Pee11g042536, Pee03g013164* and *Pee03g013165*) were down-regulated at day 3, with expression decreasing as nitrogen levels increased, but was up-regulated at day 21, with the highest expression in the control group. In contrast, glutamate synthase gene (*Pee09g035608*) was up-regulated at day 3 and down-regulated at day 21, showing the lowest expression in the control group. Glutamate decarboxylase genes (*Pee07g029810, Pee07g029812, Pee08g033055, Pee07g029811, Pee10g038424* and *Pee07g029807*) were up-regulated at day 3 and down-regulated at day 21, while genes *Pee07g026510* and *Pee02g009915* exhibited the opposite trend. Glutamate synthase gene (*Pee09g035608*) was up-regulated at day 3 and down-regulated at day 21, with the control group consistently showing the highest expression. The amidase gene was up-regulated in the control group at day 3 but significantly reduced in the high nitrogen group, and remained relatively highly expressed in the control group at day 21.

### 3.5 The correlation between phenotype and genes

**Figure 5:**
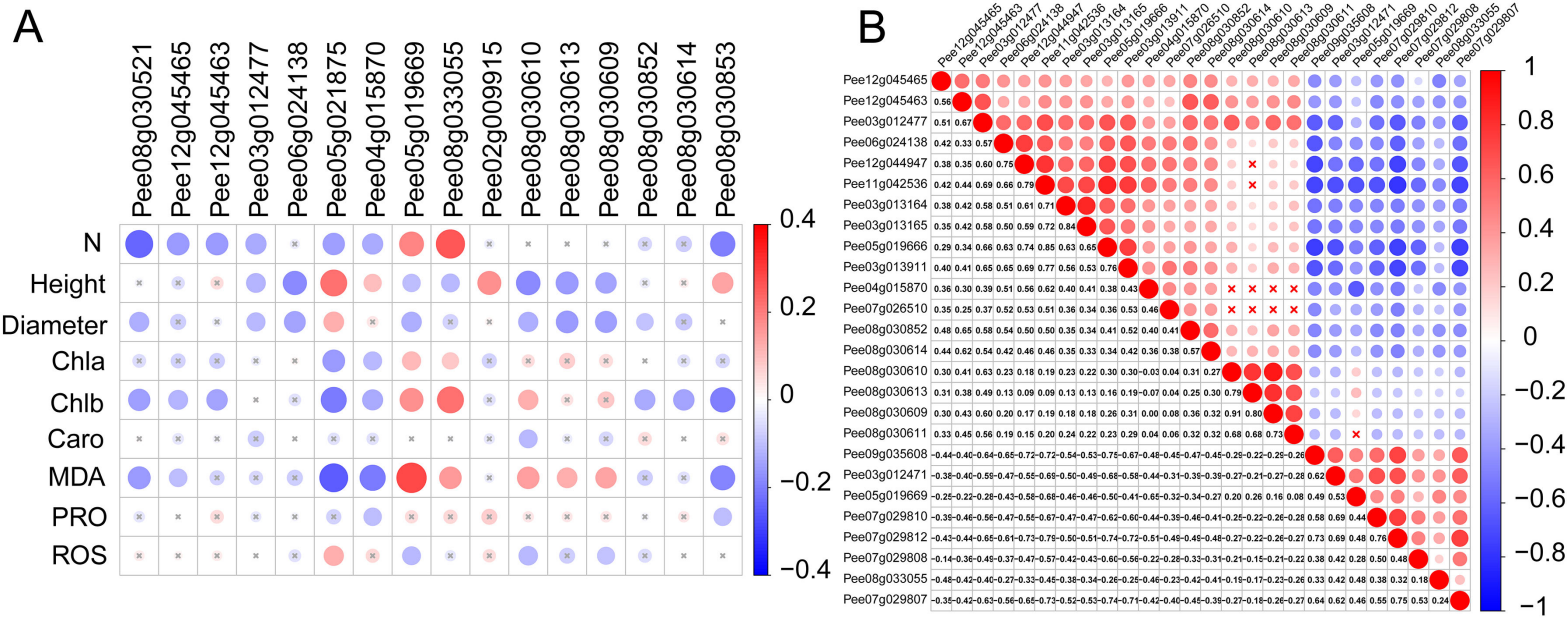
Correlations between phenotypes and genes, and among the selected genes. Genes with non-significant correlations (*p* > 0.05) were excluded. **(A)** Correlations between the determined phenotypes and the selected genes. The phenotypes include the contents of nitrogen (N), chlorophyll a (Chla), chlorophyll b (Chlb), and carotenoids (Caro) in needles, as well as seedling height (height) and ground diameter (diameter). **(B)** Correlations among the 28 selected genes. Eight genes with non-significant correlations were excluded from the initial 36 selected genes, x denotes the significance of the correlation between gene and phenotype or between genes, with *p* > 0.05.

Pearson correlation analysis was performed to assess the relationships between the expression levels of the 36 screened genes and the measured phenotypic traits (**Figure 5A**). Leaf nitrogen content showed a significant negative correlation with nitrate transporter gene *Pee08g030521* and a significant positive correlation with glutamate decarboxylase gene *Pee08g033055*. Seedling height was significantly negatively correlated with formamidase gene *Pee06g024138* and nitrate transporter gene *Pee08g030610*, but significantly positively correlated with NADP-specific glutamate dehydrogenase gene *Pee05g021875*. Chlorophyll b content was significantly positively correlated with glutamate dehydrogenase gene *Pee08g033055*, and significantly negatively correlated with NADP-specific glutamate dehydrogenase gene *Pee05g021875* and glutamate dehydrogenase gene *Pee09g035853*.

Furthermore, Pearson correlation analysis was conducted among the expression levels of the 36 screened genes (**Figure 5B**). Cluster A is a nitrogen utilization module initiated by NRT2.1, involving nitrate uptake followed by ammonium and glutamate synthesis to produce γ-aminobutyric acid (GABA), while an alternative pathway converts formamide for nitrogen utilization; Cluster B represents the glutamate-glutamine cycle module responsible for nitrogen supply; the remaining component, NRT3.1, constitutes the nitrate absorption and transport module. Overall, genes *Pee08g030521* (NRT2.1), *Pee12g045465* (NRT2.1), *Pee12g045463* (NRT2.1), *Pee03g012477* (NR), *Pee06g024138* (FMD), *Pee12g044947* (FMD), *Pee11g042536* (GLSF), *Pee03g013164* (GLSF), *Pee03g013165* (GLSF), *Pee04g015870* (NADP-GDH), *Pee05g019666* (GS), *Pee07g026510* (GAD), *Pee03g013911* (NLP3), *Pee08g030852* (NRT3.1), and *Pee08g030614* (NRT3.1) formed Cluster A, exhibiting significant positive correlations with each other. Genes *Pee09g035608* (GLT), *Pee03g012471* (GDH), *Pee05g019669* (GS), *Pee07g029810* (GAD), *Pee07g029812* (GAD), *Pee07g029808* (GAD), *Pee08g033055* (GAD) and *Pee07g029807* (GAD) formed Cluster B, also showing significant positive correlations within the group. Notably, significant negative correlations were observed between Cluster A and Cluster B. Additionally, genes *Pee08g030610* (NRT3.1), *Pee08g030613* (NRT3.1), *Pee08g030609* (NRT3.1) and *Pee08g030611* (NRT3.1) demonstrated significant positive correlations among themselves.

### 3.6 Weighted co-expression network analysis

**Figure 6:**
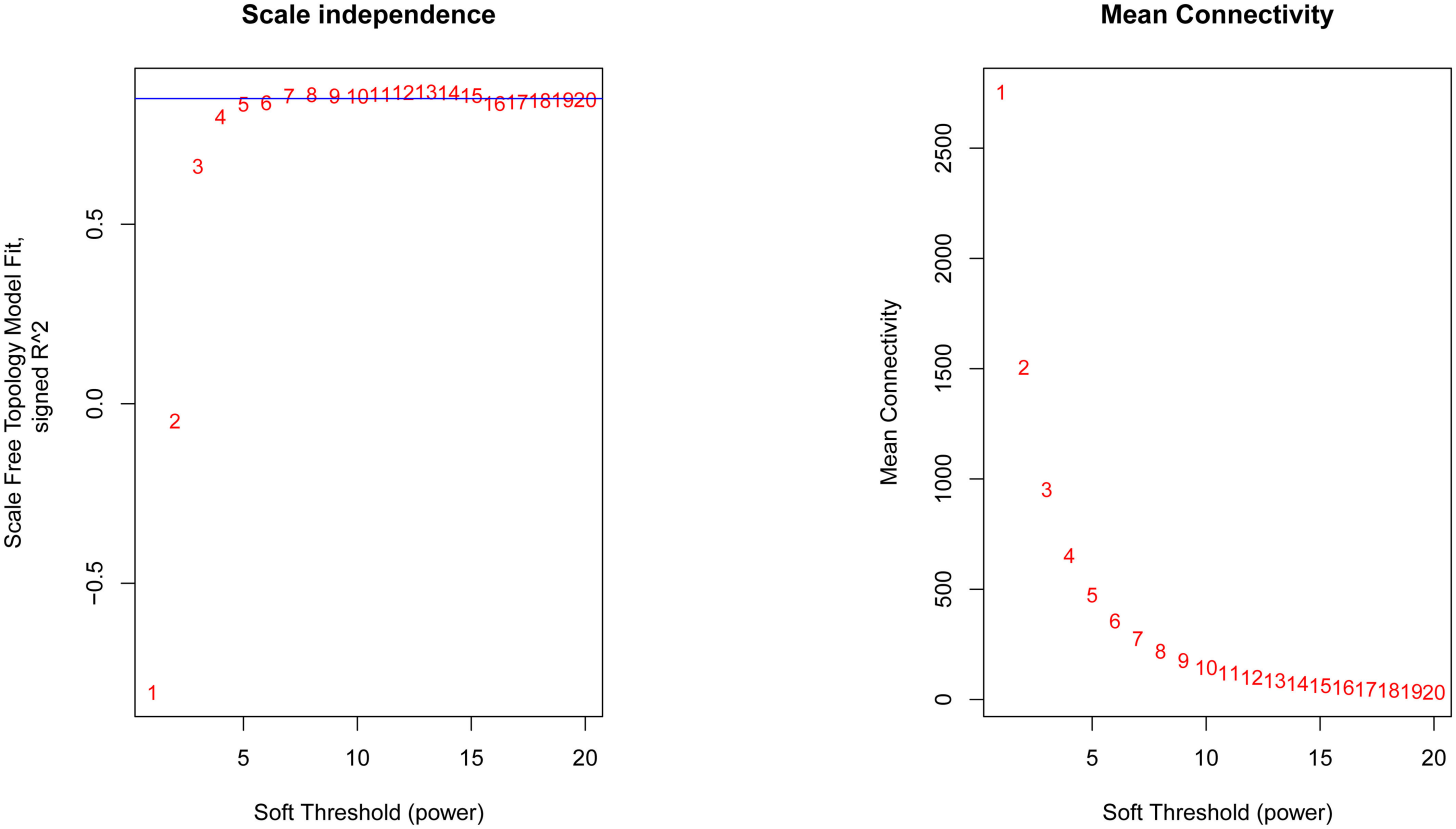
Network topology was analyzed under various soft threshold powers (β values). The blue dashed line indicates the threshold of 0.9.

A weighted gene co-expression network was built using the FPKM expression values of 7000 genes. As presented in **Figure 6**, when the soft threshold parameter *β* was determined to be 7, the scale-free topology model achieved an R^2^ value greater than 0.9, accompanied by an average connectivity close to zero. These results verified that the network satisfied the scale-free characteristics. Therefore, *β* = 7 was chosen as the optimal soft threshold for subsequent network construction.

**Figure 7:**
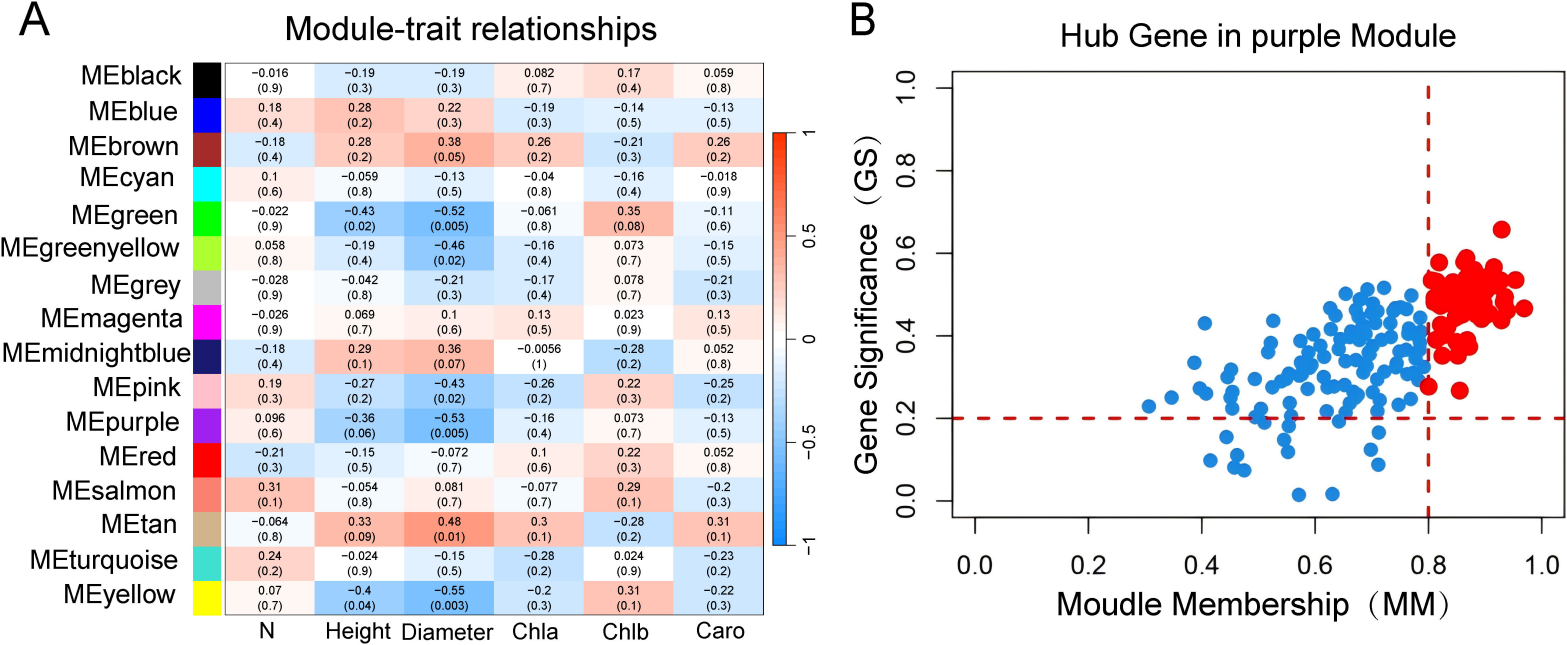
**(A)** Module–trait association analysis. Each row represents a co-expression module, while each column stands for a phenotypic trait. The correlation coefficient and corresponding p-value are displayed within each grid. A red color signifies a strong positive correlation, whereas a blue color represents a strong negative correlation. **(B)** Scatter plot illustrating gene distribution within the purple module.

**Figure 8:**
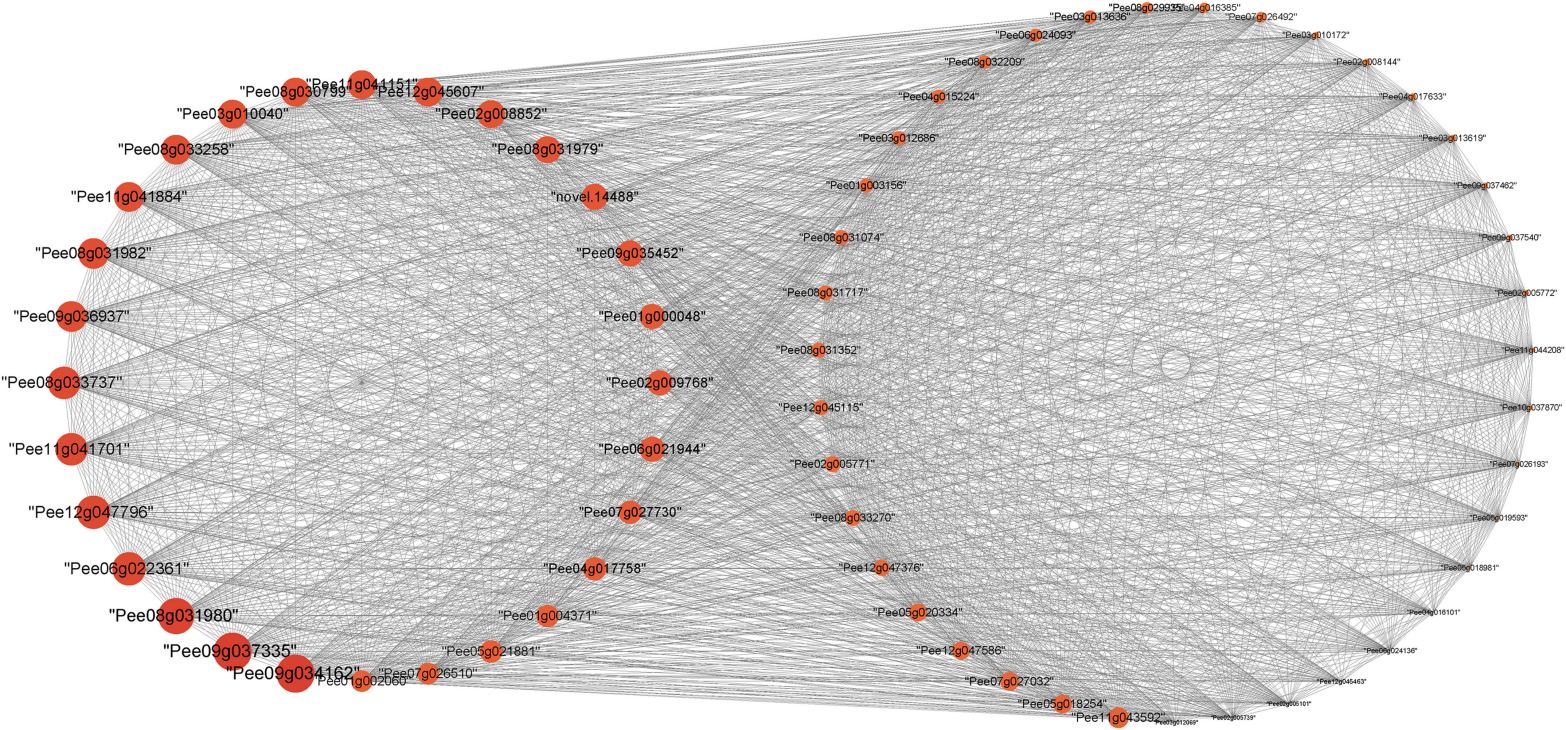
Interaction network of 63 hub genes derived from the purple module (MEpurple), visualized via Cytoscape. The gene label size is positively correlated with the node degree value.

The tan, yellow, and purple modules comprised 94, 356, and 184 genes, respectively (**Supplementary Table 1**). All three modules exhibited strong correlation with ground diameter. Functional annotation revealed that the tan module was enriched for membrane and channel-related proteins, the yellow module for transcription-related proteins, and the purple module for proteins involved in amino acid hydrolysis and glucose utilization(**Figure 7A**). Based on this functional characterization, the purple module was selected for subsequent analysis. Based on differential expression across nitrogen treatments and time points, candidate hub genes (**Supplementary Table 2**) were identified using thresholds of Gene Significance (GS) > 0.2 and Module Membership (MM) > 0.8 (**Figure 7B**). This screening process yielded 63 hub genes within the purple module, whose network relationships (**Supplementary Table 3**) were visualized using Cytoscape (**Figure 8**).

Among these 63 hub genes, *Pee04g017758, Pee11g041701, Pee11g041151* and gene *Pee07g026510* were annotated to amino acid metabolic pathways. Furthermore, gene *Pee07g026510* exhibited a degree value greater than 63. This gene is responsible for converting L-glutamate to GABA, which is subsequently converted to succinate, entering the citric acid cycle and contributing to the synthesis of other amino acids.

### 3.7 RT-qPCR results

**Figure 9:**
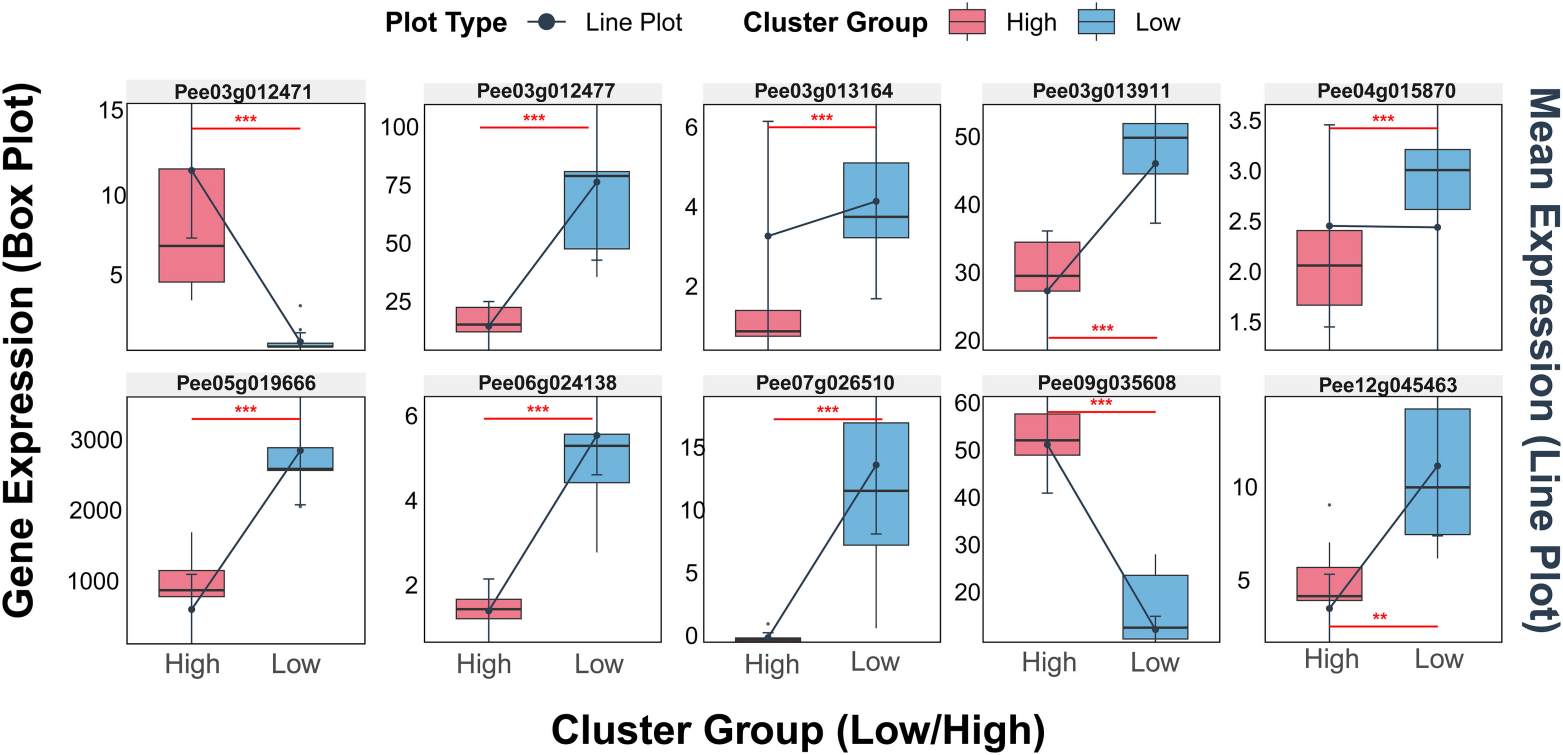
RT-qPCR verification of 10 candidate genes involved in nitrate uptake and utilization. Relative expression levels from RT-qPCR using UBI as the internal reference gene are shown on the left y-axis. Normalized expression levels from RNA-seq (log_2_(FPKM + 1)) are presented as lines on the right y-axis. Low indicates the group with smaller ground diameter, and High indicates the group with larger ground diameter. The functions of these genes are as follows: *Pee12g045463*: high-affinity nitrate transporters; *Pee03g012477*: nitrate reductase; *Pee03g013164*: ferredoxin- dependent glutamate synthase; *Pee09g035608*: glutamate synthase; *Pee03g012471*: glutamate dehydrogenase; *Pee04g015870*: NADP-specific glutamate dehydrogenase; *Pee05g019666*: Glutamine synthetase; *Pee07g026510*: glutamate decarboxylase; *Pee03g013911*: Omega-amidase; *Pee06g024138*: formamidase. “**” indicates a significance level of *p* < 0.01, and “***” indicates a significance level of *p* < 0.001.

**Figure 10:**
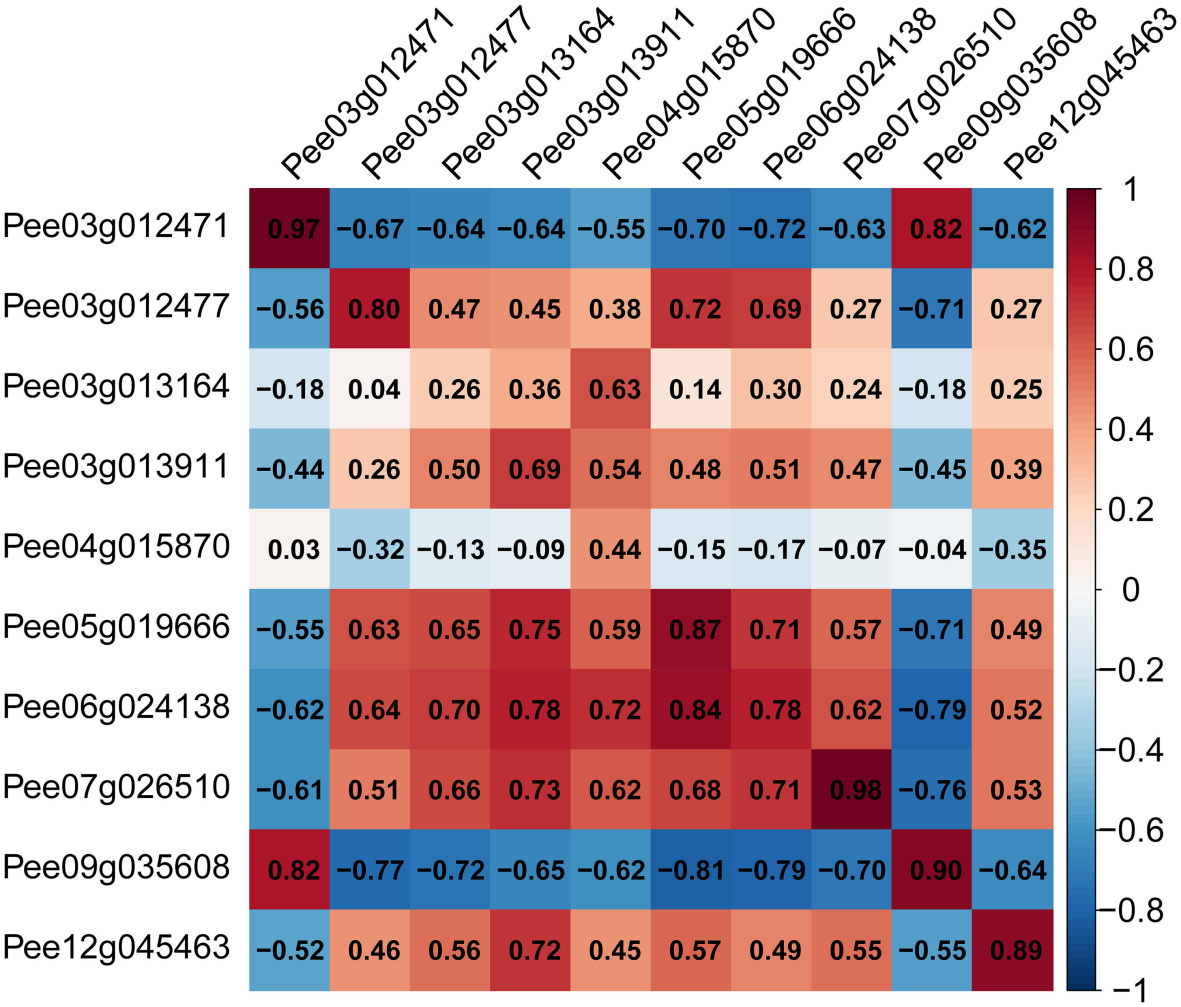
Heat map of the correlation between RNA sequencing results and RT-qPCR. The x-axis is the result of RNA sequencing, and the y-axis is the result of RT-qPCR.

Ten highly expressed DEGs related to nitrogen uptake and utilization were selected. RT-qPCR was performed using nine samples with the largest ground diameter and nine samples with the smallest diameter to validate expression profiles identified by RNA-seq (**Table 2**). Expression patterns of these ten genes from RNA-seq and RT-qPCR are shown in **Figure 9**. Except for *Pee04g015870*, the expression trends were highly consistent between RNA-seq and RT-qPCR, confirming the reliability of the transcriptome data. The descriptive statistics of the expression of 10 genes in the sample are shown in **Table 3**, and the correlation between sequencing results and RT qPCR is shown in **Figure 10**. In the sequencing results, except *Pee03g013164*, the correlation between the two results of other genes was very high.

**Table 2:** Primers used for RT-qPCR.

Gene id	Forward (5′to 3′)	Reward (3′to 5′)
Pee03g012471	GTTCCCTTGGTAGGGATGCT	CCACGACCTTTCCACCTTTC
Pee03g012477	AAAGCGGATTCTCCCAGACA	TCCCACACCACTTTCTCCTC
Pee03g013164	TTCGCCTATGAGGATGCAGT	AATGATTCACGTGCCAGCTC
Pee03g013911	AGGGCGTATTGGGATTGGAA	CCCTGGCTCTCTGTAGTAGC
Pee04g015870	GTTACCGTGTCCAGTTCAGC	TGCTCCAGTCCCAGGAATTT
Pee05g019666	ATGGGACTGCTCTTCCTTCC	GTTCAAGCCCGTACCATGTC
Pee06g024138	CAGGTGTTCGATTTCCAGGT	TGCCTCCCTTGCATTCCATA
Pee07g026510	GCTTGAGTGGGACTTTCGAC	CCGGAAGATCTTGCTTGGTG
Pee09g035608	ATGAGTTCTTGGCTGAGGCT	TTTCCTCCCGTCGAGTTTCA
Pee12g045463	GCTTCATCGCTACATTCGCT	TCACACAGACCTCCCATCAC
Pe-UBI	GATTTATTTCATTGGCAGGC	AGGATCATCAGGATTTGGGT

**Table 3:** Descriptive statistical analysis of RT-qPCR.

Gene	Cluster	Mean	SE	SR	Med	Min	Max
Pee03g012471	Low	0.803	0.884	0.295	0.425	0.154	2.941
Pee03g012471	High	8.564	4.675	1.558	6.627	3.277	15.452
Pee03g012477	Low	66.107	24.755	8.252	77.384	34.048	108.345
Pee03g012477	High	14.452	6.777	2.259	13.552	2.295	23.14
Pee03g013164	Low	4.468	1.277	0.426	3.926	3.073	6.766
Pee03g013164	High	1.224	0.542	0.181	1.05	0.562	2.04
Pee03g013911	Low	49.01	5.343	1.781	50.818	39.877	55.795
Pee03g013911	High	30.717	5.575	1.858	30.492	19.473	36.485
Pee04g015870	Low	2.975	0.446	0.149	3.001	2.437	3.658
Pee04g015870	High	2.002	0.503	0.168	2.054	1.208	2.707
Pee05g019666	Low	2903.61	425.704	141.901	2768.36	2236.477	3792.977
Pee05g019666	High	1133.416	490.924	163.641	1054.265	288.78	1871.654
Pee06g024138	Low	4.896	1.084	0.361	5.207	2.692	6.386
Pee06g024138	High	1.305	0.383	0.128	1.35	0.556	1.873
Pee07g026510	Low	12.029	6.545	2.182	12.107	1.161	19.671
Pee07g026510	High	0.351	0.469	0.156	0.245	0	1.506
Pee09g035608	Low	17.393	7.203	2.401	13.911	10.718	29.385
Pee09g035608	High	53.895	6.287	2.096	53.495	42.186	62.864
Pee12g045463	Low	10.592	3.715	1.238	9.946	6.14	14.853
Pee12g045463	High	4.8	2.192	0.731	4.11	1.606	9.004

## 4 Discussion

### 4.1 Core transcription factors function as key regulators of the nitrogen response in slash pine

A total of 2353 differentially expressed genes were identified. Among them, significant differential expression of members from transcription factor families such as C3H, bHLH, and MYB suggests these transcription factors may play core roles in the transcriptional regulatory network of nitrogen uptake and utilization in slash pine. As key regulators of gene expression, transcription factors can coordinately activate or repress nitrogen metabolism-related pathways by binding to cis-elements in the promoters of downstream target genes (Konishi and Yanagisawa, 2013; Fan et al., 2020; Feng et al., 2022), which is consistent with the co-expression pattern between transcription factor genes and nitrogen transport/assimilation genes observed in this study.

As one of the core DEGs in this study, the differential expression pattern of C3H transcription factors is highly synchronized with the nitrogen response process of slash pine Han noted that the C3H gene family is widely involved in plant development, signal transduction, and biotic/abiotic stress responses (Han et al., 2021). Its members regulate gene expression through protein-protein interactions or binding to specific cis-elements in target gene promoters. In Cucumis melo (muskmelon), the CmC3H gene can regulate plant adaptability by responding to stresses such as drought and fusarium wilt.

The functions of bHLH genes in Arabidopsis thaliana have been well documented (Hao et al., 2021). Members of the bHLH transcription factor family account for a significant proportion of DEGs in this study, which is consistent with the versatility of this family in plant nitrogen response and growth and development. Previous studies have shown that bHLH transcription factors affect plant nitrogen use efficiency by regulating the expression of nitrogen transporter genes, participating in nitrogen signal transduction, and mediating hormone crosstalk (Jia et al., 2022).

The differential expression of MYB transcription factor family members further enriches the complexity of the nitrogen response regulatory network in slash pine. Existing studies have confirmed that members of the R2R3-MYB subfamily within the MYB family are widely involved in plant nitrogen stress responses. For example, Chrysanthemum morifolium CmMYB42 significantly improves low-nitrogen stress tolerance in transgenic plants by upregulating the expression of NRT family genes and enhancing nitrogen assimilatory enzyme activity (Ma et al., 2023). These MYB members may function through similar mechanisms: first, directly binding to the promoter regions of key nitrogen metabolism genes such as NRT2.1 and GS to promote their expression and enhance nitrogen uptake and assimilation; second, regulating the expression of secondary cell wall synthesis genes (e.g., HCT, ADT6) to promote root development and lignin synthesis, thereby expanding the nutrient absorption surface area (Ma et al., 2023). The coordinated differential expression of these transcription factors indicates that the nitrogen response of slash pine is a complex regulatory process involving multiple transcription factor families, and their specific regulatory targets and interaction networks require further verification.

### 4.2 Slash pine adapts to nitrogen environments via differential expression of NRT and nitrogen assimilation genes

Nitrate transporters are core carriers for plants to absorb nitrate from the soil, and their expression levels and activities directly determine plant nitrogen uptake efficiency (Krapp, 2015). The significant differential expression of NRT2.1 and NRT3.1 in this study is highly matched to the nitrate uptake demand of slash pine under different nitrogen concentrations. As representatives of high-affinity nitrate transporters, the NRT2 family can account for 95% of nitrate uptake under low-nitrogen conditions (Wang et al., 2012). The elevated expression of NRT2.1 in the low-nitrogen treatment group suggests that *Pinus elliottii* may enhance nitrate acquisition capacity in nitrogen-limited environments through upregulated NRT2.1 expression, implying a potential role as a key contributor to nitrate transport function within the NRT2 family in needles. Members of the NRT3 family (NRT3.1), as auxiliary factors of NRT2 transporters, regulate their activity and stability by forming complexes with NRT2 (Yong et al., 2010). The coordinated differential expression of NRT3.1 and NRT2.1 in this study further supports their functional relevance in nitrate uptake of slash pine (Kiba et al., 2012).

Notably, this study also identified differential expression of other NRT3 family members such as NRT3.2, which is consistent with the expansion characteristic of the NRT3 family in gymnosperms (Okamoto et al., 2006). Previous studies have found that the expansion of the NRT3 family in conifers may be an important evolutionary strategy for their adaptation to different nitrogen environments (Castro-Rodríguez et al., 2017). The differential expression patterns of NRT3.1 and NRT3.2 in this study suggest that slash pine flexibly regulates nitrate uptake efficiency to adapt to nitrogen concentration fluctuations through the differential expression of NRT3 family members.

Nitrogen assimilation is the core process of converting absorbed nitrate into plant-available organic nitrogen (e.g., amino acids, proteins), and its key enzymes include NR (Gao et al., 2019), GS (Wei et al., 2020), GOGAT (Masclaux-Daubresse et al., 2006), GDH (Melo-Oliveira et al., 1996), etc. The differential expression of these enzyme-encoding genes in this study reflects the adaptive regulation of nitrogen assimilation efficiency in slash pine in response to changes in nitrogen concentration.

Nitrate reductase, as the first key enzyme in nitrogen assimilation, is responsible for reducing nitrate to nitrite, and its activity directly affects the rate of nitrogen assimilation (Wilkinson and Crawford, 1993).The high expression of NR gene in the low-nitrogen treatment group aligns with the increased physiological demand for nitrate assimilation under nitrogen-limited conditions. In contrast, the downregulation of NR gene in the high-nitrogen treatment likely reflects an adaptive strategy in slash pine to modulate nitrate uptake under excessive nitrogen supply, which is consistent with observations in *Matricaria chamomilla* (Repčák et al., 2014) and *Azolla spp* (Zheng et al., 2022). The GS/GOGAT cycle, composed of glutamine synthetase (GS) and glutamate synthase (GOGAT), is the core pathway of plant nitrogen assimilation, responsible for converting ammonium nitrogen into glutamate and glutamine (Konishi et al., 2016). The differential expression of GS isozymes (cytosolic isozyme, nodule isozyme) and GOGAT (GLSF, GLT1) genes in this study reflects the tissue specificity and functional differentiation of nitrogen assimilation in slash pine.

Glutamate dehydrogenase (GDH), as a supplementary pathway to the GS/GOGAT cycle, plays an important role in nitrogen metabolism balance. Its NADP-specific isozyme (NADP-GDH) can convert excess ammonium nitrogen into glutamate when nitrogen is sufficient, and participate in glutamate catabolism to recycle nitrogen when nitrogen is deficient (Masclaux-Daubresse et al., 2005). The differential expression of GDH and NADP-GDH genes in this study suggests that slash pine maintains nitrogen metabolism balance to adapt to different nitrogen environments by regulating the activity of the GDH pathway. In addition, the differential expression of glutamate decarboxylase (GAD) and ω-amidase (NLP3) further enriches the regulatory network of nitrogen metabolism in slash pine: GAD participates in nitrogen storage and transport by converting glutamate into γ-aminobutyric acid (GABA) (Majumdar et al., 2016), while NLP3, as a nitrogen-responsive transcription factor, can directly regulate the expression of nitrate transporter and assimilatory enzyme genes (Zhang et al., 2022). Their synergistic effect may play a key regulatory role in the nitrogen response of slash pine.

The present study demonstrates that nitrogen availability modulates oxidative stress responses in *Pinus elliottii* seedlings in an organ-specific manner. ROS content was lowest across all organs under high-nitrogen conditions, confirming that both low-nitrogen and control groups experienced oxidative stress (Li et al., 2020; Ma et al., 2025). Notably, MDA responses differed between aboveground and belowground organs: while leaves and stems exhibited the lowest MDA content under high-nitrogen treatment as expected, roots displayed elevated MDA levels. This pattern may be attributed to direct root exposure to nitrogen causing localized oxidative stress, accelerated root growth leading to faster senescence, and preferential allocation of antioxidant resources to photosynthetic organs. The elevated proline content in roots and stems under high-nitrogen conditions likely reflects enhanced amino acid synthesis driven by nitrogen availability rather than stress response.

The contrasting pattern of MDA accumulation in roots under high-nitrogen conditions—contrary to the decline observed in leaves and stems—suggests a potential interplay between accelerated root growth and localized senescence processes. While increased MDA content in roots is commonly associated with enhanced lipid peroxidation, this observation may imply a complex physiological response in which nitrogen-driven root proliferation and metabolic demand potentially outpace the protective capacity of antioxidant systems. This scenario aligns with studies indicating that elevated antioxidant enzyme activities (e.g., SOD, POD, CAT) under increased nitrogen availability can mitigate oxidative damage and reduce MDA levels in shoots (Shah et al., 2024). However, in roots, the concurrent dynamics of rapid growth, resource allocation, and cellular turnover may introduce additional stressors, potentially overwhelming antioxidant defenses. This hypothesis is tentatively supported by findings in high-saline habitats, where root MDA accumulation co-occurred with elevated SOD and POD activities, implying a trade-off between metabolic activity and oxidative burden (Jiang et al., 2024). Notably, the current study lacks direct morphological evidence (e.g., root branching density, cell death rates) to confirm this mechanistic link; thus, the proposed relationship between nitrogen-induced root growth, senescence, and MDA accumulation remains a plausible yet unverified hypothesis.

### 4.3 *Pee07g026510* potentially orchestrates nitrogen metabolism to modulate diameter growth in slash pine

WGCNA revealed a strong correlation between ground diameter and module gene expression. Network visualization of 63 hub genes showed that *Pee07g026510* exhibited high connectivity with other genes, indicating it may play a core regulatory role in Diameter growth of slash pine under nitrogen stress. As a key agronomic trait for the growth and development of slash pine, Diameter directly reflects plant nitrogen use efficiency and productivity.

Nitrogen, an essential element for plant growth and development, regulates plant growth by affecting processes such as photosynthesis and biomass accumulation (Chen et al., 2021). The co-expression pattern between Diameter growth (a key manifestation of biomass accumulation) and nitrogen metabolism genes confirms the direct regulatory role of nitrogen metabolism in slash pine growth. Previous studies have shown that GATA transcription factors in poplar significantly improve plant Diameter and biomass by enhancing nitrogen uptake and assimilation efficiency (Shen et al., 2022). The Diameter-related module identified in this study contains a large number of key nitrogen metabolism genes (e.g., NRT2.1, GS, GAD), further supporting the close association between nitrogen metabolism and Diameter growth. Under low-nitrogen conditions, the upregulation of genes such as NRT2.1 and GS in slash pine suggests enhanced nitrogen acquisition and assimilation, potentially contributing to the material and energy foundation for Diameter growth. Conversely, under high-nitrogen conditions, moderate expression of nitrogen metabolism-related genes may help mitigate the risk of growth inhibition associated with excessive nitrogen accumulation. As a hub gene in the co-expression network, *Pee07g026510* exhibits high connectivity, implying a putative role in coordinating regulatory crosstalk between nitrogen metabolism and Diameter growth pathways. The reaction catalyzed by GAD—converting glutamate to γ-aminobutyric acid (GABA)—is not only an important branch of plant nitrogen metabolism but also participates in multiple physiological processes such as plant growth and development and stress response (Fait et al., 2011; Al-Quraan et al., 2019). In Arabidopsis thaliana, overexpression of GAD genes can affect root development and biomass accumulation by regulating GABA content (Li et al., 2018).

The strong correlation between *Pee07g026510* and diameter trait in this study suggests it may regulate slash pine growth through the following mechanisms: By regulating the synthesis and accumulation of GABA, it participates in nitrogen storage and transport, providing a stable nitrogen supply for Diameter growth. Through GABA-mediated signal transduction, it regulates the balance of hormones such as auxin and cytokinin, thereby affecting vascular tissue development and cell proliferation, and ultimately regulating diameter growth. As a crosstalk node between nitrogen metabolism and carbon metabolism, it provides energy and material support for Diameter growth by regulating the metabolic balance between GABA and carbohydrates (Fait et al., 2011; Kanwal and Incharoensakdi, 2019).In addition, the high connectivity between *Pee07g026510* and other nitrogen metabolism genes (e.g., NRT2.1, GS) indicates it may regulate the expression of these genes to achieve coordinated regulation of nitrogen metabolism and growth. In this study, the hub gene *Pee07g026510* may affect slash pine Diameter growth through two aspects by regulating GAD expression: Under high-nitrogen conditions: GAD catalyzes the conversion of glutamate to GABA, which can consume excess ammonium nitrogen and avoid its toxicity to cells (Jung et al., 2023). Meanwhile, GABA may promote vascular tissue development by regulating the polar transport and signal transduction of auxin (Chen et al., 2023), thereby increasing Diameter. Under low-nitrogen conditions: *Pee07g026510* may downregulate GAD expression, reducing the conversion of glutamate to GABA to prioritize the nitrogen assimilation efficiency of the GS/GOGAT cycle. This provides more nitrogen for needle photosynthesis and indirectly supports Diameter growth.

Furthermore, the high connectivity between *Pee07g026510* and nitrogen metabolism genes such as NRT2.1 and GS suggests it may act as a “hub gene” to integrate nitrogen uptake, assimilation, and growth signals. This finding not only conforms to the general rule of gene co-expression networks regulating growth and metabolism but also provides a direct target for improving nitrogen use efficiency and growth performance of slash pine through gene editing.

### 4.4 Slash pine nitrogen metabolism genes exhibit synergistic/antagonistic regulatory relationships

Pearson correlation analysis classified 36 key genes into different groups. Group A (NRT2.1, NR, FMD, GLSF, NADP-GDH, GS, GAD, NLP3, NRT3.1) showed significant positive correlations within the group. Group B (GLT1, GDH1, GS, GAD) also exhibited significant positive correlations internally, while a significant negative correlation was observed between Groups A and B. Members of the NRT3 family (NRT3.1, NRT3.2) showed significant positive correlations among themselves. These results clearly reveal the regulatory network structure and synergistic/antagonistic relationships of nitrogen metabolism in slash pine.

Group A includes key nitrate transporters (NRT2.1, NRT3.1), core nitrogen assimilation enzymes (NR, GLSF, NADP-GDH, GS), and metabolic regulatory proteins (GAD, NLP3, FMD). The significant positive correlations within Group A indicate strong synergistic effects of these genes in nitrogen metabolism. NRT2.1 and NRT3.1, key components of the nitrate transport system, exhibit elevated expression under conditions favoring nitrate uptake, suggesting enhanced nitrate transport into cells. This upregulation potentially facilitates the supply of substrates for subsequent nitrogen assimilation pathways. NR, the key enzyme in the first step of assimilation, reduces nitrate to nitrite, supplying precursors for subsequent enzymatic reactions such as those catalyzed by GLSF (ferredoxin-dependent glutamate synthase) and the cytosolic isozyme of GS. NADP-GDH and NLP3 ensure efficient assimilation by regulating nitrogen metabolism balance. The core regulatory gene *Pee07g026510* provides signal and material support for nitrogen uptake and assimilation through regulating GABA metabolism. This synergistic expression pattern conforms to the linear pathway characteristics of nitrogen metabolism (“nitrate uptake → ammonium assimilation → metabolic balance”). Synchronous upregulation or downregulation of genes in each link ensures the efficient and coordinated progression of nitrogen metabolism.

Group B comprises GLT1, GDH1, GS, and GAD. Its significant internal positive correlations and significant negative correlation with Group A suggest that Group B may serve as a complementary and antagonistic regulatory pathway to the core nitrogen metabolism pathway mediated by Group A.GLT1, another member of the GOGAT family, may have tissue- or developmental stage-specific functional differentiation from GLSF in Group A, responsible for glutamate synthesis under specific conditions (Forde and Lea, 2007).GDH1, an isozyme of the GDH family, may function when the activity of NADP-GDH in Group A is limited or participate in nitrogen remobilization (Eprintsev and Anokhina, 2023).The negative correlation between Groups A and B may reflect the dynamic balance regulation of nitrogen metabolism in slash pine: when the core pathway of Group A is activated, the complementary pathway of Group B is inhibited to avoid metabolic redundancy; when the Group A pathway is limited, the Group B pathway is activated to maintain basic nitrogen metabolism needs. This antagonistic relationship ensures the flexibility and stability of nitrogen metabolism in slash pine under different nitrogen environments.

The significant positive correlations among NRT3 family members (*Pee08g030610* [NRT3.1], *Pee08g030613* [NRT3.2], *Pee08g030609* [NRT3.1]) further confirm the synergistic role of the NRT3 family in nitrate uptake of slash pine. Previous studies have shown that NRT3 family members form heterologous complexes with the NRT2 family to enhance transport activity and stability (Wang et al., 2021). The synergistic expression of NRT3 family members in this study may function through the following mechanisms: Multiple NRT3 members form polycomplexes with NRT2.1, significantly improving nitrate transport efficiency under low-nitrogen conditions. Differential expression of different NRT3 members in time or space ensures the continuity and stability of nitrate uptake. NRT3 family members participate in nitrogen signal transduction through synergistic regulation, transmitting nitrate concentration signals to downstream transcription factors to achieve coordinated regulation of nitrogen uptake and assimilation (Jia et al., 2023).This synergistic expression pattern is an important molecular strategy for slash pine to adapt to nitrogen concentration fluctuations, and also provides new insights into the functional evolution of the NRT3 family in gymnosperms.

When compared with a broader range of plant species, the transcriptional regulatory characteristics of *Pinus elliottii* in response to nitrogen show both conserved and species-specific patterns. In Eucalyptus grandis (Li et al., 2024), the NPF gene family is regulated by nitrogen availability and enhances tolerance to nitrogen stress by modulating nitrogen transport and redistribution, which is consistent with the regulatory roles of DEGs involved in nitrogen metabolism pathways identified in this study. In poplar (Luo et al., 2015; Lu et al., 2019), transcriptome reprogramming dominates morphological and physiological adaptation to nitrogen starvation or excess, with enriched GO terms related to development, nitrogen metabolism, and hormone stimulus in roots and leaves, which is consistent with the GO enrichment patterns of DEGs in response to nitrogen in *Pinus elliottii*. In Yellowhorn (Li et al., 2023)., plant hormone signal transduction pathways are upregulated under low-nitrogen stress, which is consistent with the transcription factor-mediated regulatory logic observed in this study. Notably, the mechanism by which the hub gene *Pee07g026510* affects growth by regulating GAD expression and the efficiency of the GS/GOGAT cycle identified in this study has not been reported in Eucalyptus grandis, poplar, or Yellowhorn, thus representing a species-specific feature of nitrogen response in *Pinus elliottii*.

## 5 Conclusions

This study identified the core transcriptional regulatory targets of nitrogen response in slash pine. A total of 2353 differentially expressed genes were identified, among which transcription factor (TF) families such as C3H, bHLH and MYB were significantly enriched, and all these TFs have clear associations with plant nitrogen uptake and utilization. The nitrogen uptake and utilization pathway contains 36 DEGs encoding 10 types of proteins. This study also uncovered the associations and synergistic regulatory patterns between nitrogen metabolism genes and growth traits of slash pine. WGCNA analysis showed a strong correlation between ground diameter of slash pine and module gene expression, and the hub gene *Pee07g026510* exhibited high connectivity with other nitrogen metabolism genes. This confirms that *Pee07g026510* can affect Diameter growth by regulating GAD expression—specifically, generating GABA to alleviate ammonium toxicity under high-nitrogen conditions and prioritizing the efficiency of the GS/GOGAT cycle under low-nitrogen conditions. This is consistent with the theory that nitrogen deficiency inhibits biomass accumulation, providing a fundamental reference for the management and genetic improvement of slash pine plantations.

## Data Availability

The data presented in the study are deposited in the China National Center for Bioinformation (CNCB) under the accession number PRJCA052915 (https://www.cncb.ac.cn/).

## Supplementary material

Supplementary Data Sheet 1 Differentially expressed genes table.

Supplementary Table 1 WGCNA module assignments.

Supplementary Table 2 Hub gene list.

Supplementary Table 3 Network-associated data.
